# Microwave Kinetic Inductance Detector Made of Molecular Beam Epitaxy (MBE)-Grown MgB2 Film

**DOI:** 10.3390/nano14211731

**Published:** 2024-10-29

**Authors:** Ariel Roitman, Corentin Pfaff, Thomas Hauet, Avner Shaulov, Yosef Yeshurun

**Affiliations:** 1 Institute of Superconductivity, Department of Physics, Bar-Ilan University, Ramat-Gan 5290002, Israel; shauloa@mail.biu.ac.il; 2 Institute of Nanotechnology, Bar-Ilan University, Ramat-Gan 5290002, Israel; 3 Institut Jean Lamour, Université de Lorraine, Centre National de la Recherche Scientifique (CNRS), F-54000 Nancy, France; corentin.pfaff@univ-lorraine.fr (C.P.); thomas.hauet@univ-lorraine.fr (T.H.)

**Keywords:** MBE-grown superconductors, kinetic inductance detector, MgB_2_

## Abstract

We present a MgB_2_-based Microwave Kinetic Inductance Detector (MKID) featuring a quality factor Q_i_ ~ 10^5^ and noise equivalent power NEP ~ 10^−14^ $W/\sqrt{\mathrm{Hz}}$ at 2 K. In comparison to YBCO-based MKIDs, the MgB_2_ detector shows greater sensitivity to both temperature and magnetic field, a result of its two-gap nature and relatively low critical $H_{c2}$ field. Our data indicate that MgB_2_ is more advantageous for MKID applications at temperatures lower than 3 K.

## 1. Introduction 

Microwave Kinetic Inductance Detectors (MKIDs) represent a state-of-the-art technology in the field of superconducting photon detectors, offering exceptional sensitivity and multiplexing capabilities [1,2,3]. These detectors are fabricated from superconducting films lithographically patterned into a microwave LC resonance circuit. Incident photons with sufficient energy break Cooper pairs inside the superconductor, causing a change in its kinetic inductance which is observed as a shift in the resonance frequency.

Traditionally, low-$T_{c}$ superconductors, such as Al [4] and Nb [5], have been employed in the fabrication of MKIDs, owing to their well-understood superconducting properties and relatively straightforward fabrication processes. There have been limited reports of MKIDs made out of superconductors with relatively high $T_{c}$, such as YB_2_Cu_3_O_7-*δ*_ (YBCO) and ${\mathrm{MgB}}_{2}$ [6]. The motivation for exploring these materials for application in MKIDs is their potential for operation at relatively high temperatures, reducing the complexity and cost of cooling systems. An MKID made of YBCO was reported by Sato et al. [7], featuring a relatively low-quality factor of $Q_{i}$ ∼ 3000 and relatively high noise equivalent power (NEP) of ∼10^−9^ W/$\sqrt{\mathrm{Hz}}$ at 13 K. Roitman et al. [8] found better $Q_{i}$ and NEP, with values of 2.5 *×* 10^4^ and ∼10^−^^12^ W/$\sqrt{\mathrm{Hz}}$ at 10 K, respectively, using thinner YBCO films and an improved fabrication technique. MgB_2_ with a $T_{c}\;$ of around 39 K [9] offers some advantages over YBCO, such as a simple crystal structure, being composed of two abundant, low-cost elements, as well as mechanical robustness, making it easier to handle compared to the more brittle YBCO. Most of the publications on MgB_2_ relevant to its application in MKIDs [6,10,11,12,13] focus on studying temperature and field dependence of their resonance frequency, $f_{r}$, and the quality factor, $Q_{i}$. For example, Yang et al. [10] reported on lumped-element KIDs made of MgB_2_ thin films fabricated by hybrid physical–chemical vapor deposition (HPCVD) and found a high loaded quality factor, $Q_{L}$ ~ 30,000 at 7.5 K, comparable to that of lower-operating-temperature lumped-element KIDs made from superconductors such as Al and Nb.

The effect of the two gaps characterizing MgB_2_ on the microwave properties of this material was investigated by Ghigo et al. [11]. They measured the complex impedance of polycrystalline MgB_2_ films using a coplanar waveguide resonator technique. The $Q_{i}$ for their device at 5 K was ~60,000. They concluded that the temperature dependence of the penetration depth can be accounted for by an effective mean energy gap, in agreement with the predictions of Kogan [14]. Data on MgB_2_ MKIDs as a photon detector, namely, responsivity and noise equivalent power, are scarce.

In this paper, we report on an MKID made of molecular beam epitaxy (MBE)-grown MgB_2_ [15,16,17,18] and characterize it as a photon detector. The aim of using a relatively clean MBE-grown MgB_2_ was to obtain a better efficiency of Cooper pairs breaking and thus a better responsivity and lower NEP. The performance of this MKID is compared with that of an MKID made of 50 nm thick YBCO with a $T_{c}$ of around 84 K [8]. Although these MKIDs show qualitatively similar temperatures and magnetic field dependences of $f_{r}$ and $Q_{i}$, quantitatively, they are very different. The MgB_2_-based detector exhibits a much stronger decrease in the resonance frequency and the quality factor with increasing temperature or magnetic field. The strong temperature and field dependence in MgB_2_ are ascribed below to its two-gap nature and relatively lower second critical field $\;H_{c2}$, respectively.

## 2. Materials and Methods

**Figure 1:**
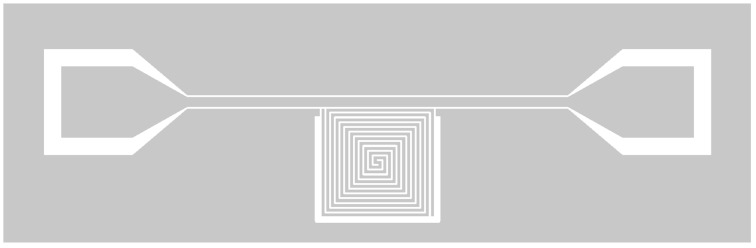
Schematic of the ${\mathrm{MgB}}_{2}$ MKID. In white: bare substrate. In gray: superconductor. The resonator linewidth is 10  μm with 10  μm between lines.

Our MgB_2_ MKID consisted of a rewound spiral λ/2 coplanar waveguide resonator coupled to a 50 Ohm transmission line, as shown in Figure 1. The device was lithographically patterned on a single-crystal 33 nm thick MgB_2_ film deposited by molecular beam epitaxy (MBE) under ultrahigh vacuum on sapphire (0001) single-crystal substrate, as described in Ref. [18]. A 5 nm thick epitaxial MgO (111) buffer layer was deposited prior to MgB_2_ in order to allow single-crystal growth of MgB_2_. A 5 nm thick capping layer of gold protected the MgB_2_ layer against oxidation. The temperature dependence of the film resistance indicated a sharp transition at $T_{c}=24.3\;K.$

The patterning process was conducted as follows. The film was coated with AZ1518 photoresist using a spin coater, followed by hot plate baking at 100 °C. Lithography was performed using a Maskless Laser Aligner (MLA150) by Heidelberg Instruments (Heidelberg, Germany) after dose calibration, followed by a photoresist development in AZ351b:$H_{2}O$ 1:4 developer. The Ar milling process was conducted in 4 s pulses, with nitrogen gas cooling in between pulses to prevent heating of the photoresist. The resist was subsequently removed using acetone to prevent any adverse effects on the resonator. 

The fabricated MKID was placed in a dedicated setup inserted into a Quantum Design (Pfungstadt, Germany) Physical Property Measurement System (PPMS). The resonance frequency, $f_{r}$, and the internal quality factor, $Q_{i}=\frac{Q_{L}}{\;\left[S_{21}\left(f_{r}\right)\right]}$, ($Q_{L}=\frac{f_{r}}{\Delta f_{3db}}$) [8], were measured using a Keysight (Santa Rosa, CA, USA) Vector Network Analyzer (VNA) connected to the detector through a combination of 20 and 30 dB attenuators at the output of the VNA and a High Electron Mobility Transistor (HEMT) amplifier and Low-Noise Amplifier (LNA) at its input. The HEMT and the 30 dB attenuator were cooled to ~5 K, while the 20 dB attenuator and the LNA were at room temperature. For the NEP measurements, the MKID was irradiated with 1064 nm light generated by an Yttrium Aluminum Garnet (YAG) laser and delivered by an optical fiber. The end of the optical fiber was positioned ~4 mm from the detector.

A magnetic field was applied perpendicular to the detector using the 9 Tesla magnet of the PPMS. In a zero-field-cooled (ZFC) procedure, the device was cooled in zero magnetic field, and then the magnetic field was changed in the superconducting phase. In the field-cooled (FC) procedure, the magnetic field was applied in the normal phase, and then the device was cooled down; the field remained unchanged in the superconducting phase.

## 3. Results

**Figure 2:**
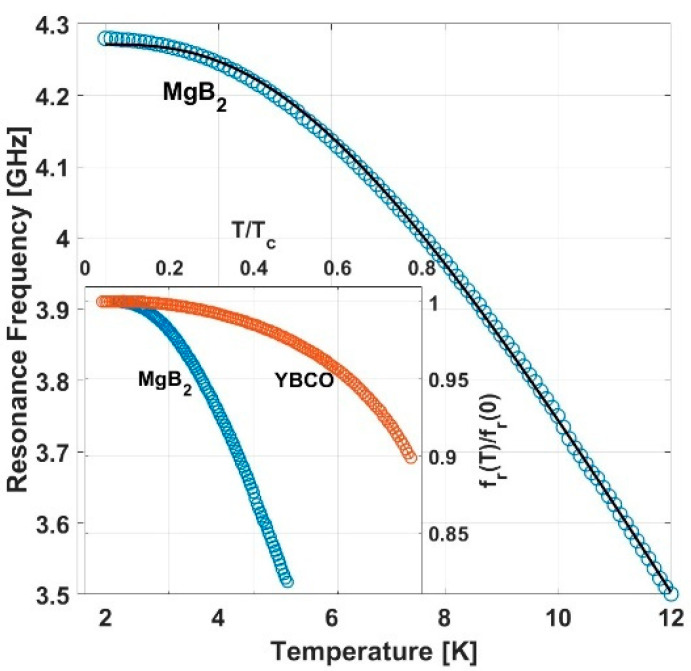
Temperature dependence of the resonance frequency, $f_{r}$, in zero magnetic field (main frame) for MgB_2_ resonator. Solid line is calculated using Equation (4). Inset: comparison of $f_{r}\left(T\right)/f_{r}\left(0\right)$ vs. $T/T_{c}$ in MgB_2_ and YBCO. The data for YBCO are based on Ref. [8].

Figure 2 presents the temperature dependence of the resonance frequency, $f_{r},$ measured in zero magnetic field. As expected, $f_{r}$ decreases with increasing temperature; however, it had a much higher rate compared to YBCO, as demonstrated in the inset in the figure, where $f_{r}\left(T\right)/f_{r}\left(0\right)$ is plotted versus *T*/*T_c_*. As discussed below, the temperature dependence of $f_{r}$ in MgB_2_ reflects the two-gap nature of this material.

**Figure 3:**
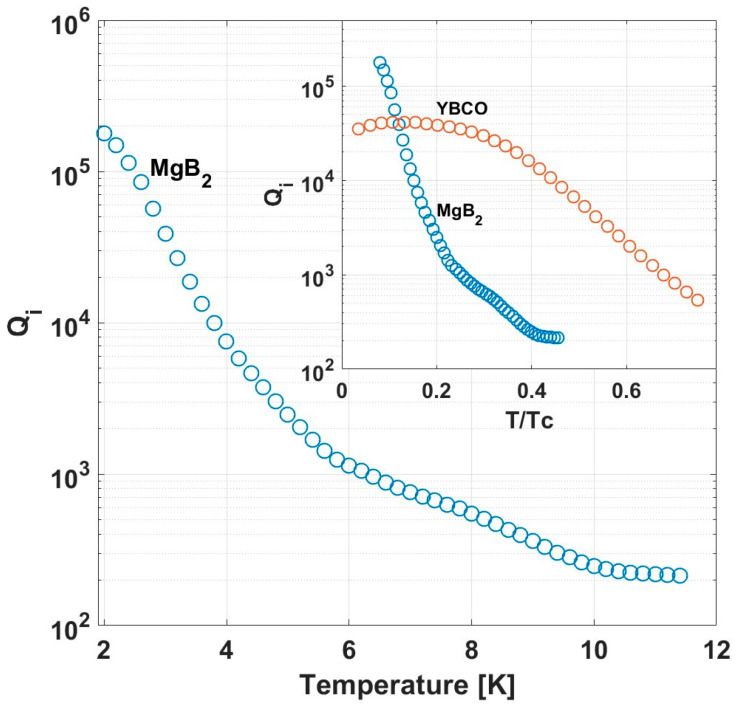
Quality factor vs. temperature for MgB_2_ in zero magnetic field (main frame). Inset: comparison of $Q_{i}\;$ vs. $T/T_{c}$ in MgB_2_ and YBCO.

The temperature dependence of the internal quality factor, $Q_{i}$, measured in zero magnetic field is shown in Figure 3. Far below $T_{c}$, a high value of $Q_{i}$, of order 10^5^, is achieved; however, it rapidly deteriorates as $T_{c}$ is approached. The inset in Figure 3 compares the behavior of $Q_{i}$ vs. $T/T_{c}$ in MgB_2_ and YBCO. Similar to the behavior of $f_{r}$, $Q_{i}$ in YBCO also decreases more moderately with increasing temperature compared to MgB_2_. Interestingly, while $Q_{i}$ in YBCO saturates at low temperatures, in MgB_2_ it continues to increase as temperature decreases, reaching values well above those of YBCO.

**Figure 4:**
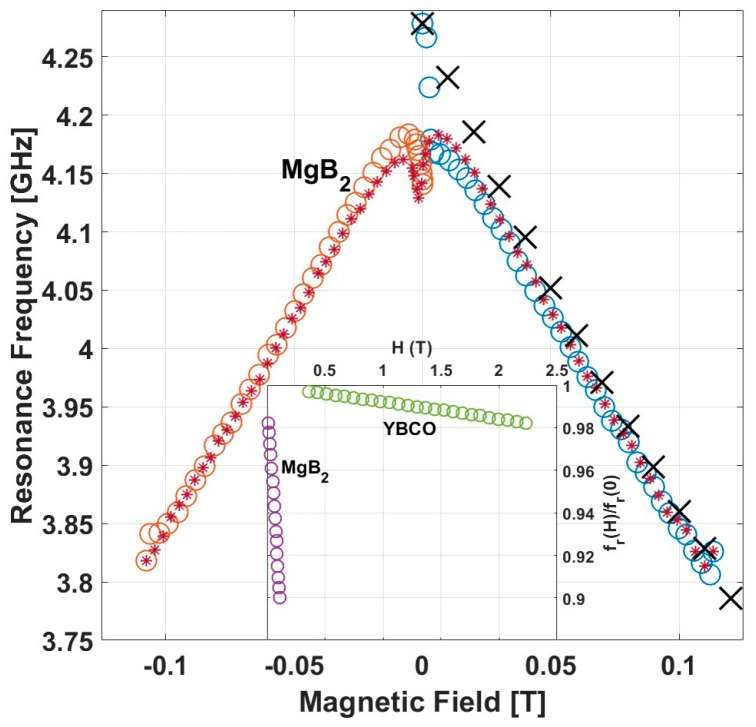
ZFC and FC measurements of the field dependence of $f_{r}$ at 2.3 K of the MgB_2_ resonator. In ZFC, the field was ramped up from 0 to 0.12 T (blue circles). It was then ramped down to −0.12 T (red stars) and back to 0 (orange circles). The FC data are denoted by black X’s. Inset: Comparison of $f_{r}\left(H\right)/f_{r}\left(0\right)$ vs. magnetic field in MgB_2_ and YBCO at $T/T_{c}≅1/6$.

Figure 4 shows ZFC and FC measurements of the field dependence of $f_{r}$ at 2.3 K with fields up to 0.12 T and down to −0.12 T and back to zero. Similar to the behavior previously observed in resonators made of other materials [8,19,20,21,22,23], MgB_2_ also exhibits an initial sharp drop in $f_{r}$ followed by an approximately linear drop with the field. Also, as previously observed, there is a substantial difference between the ZFC and FC measurements. The later shows approximately a linear decrease with the field in the entire field range in contrast to the relatively more complex ZFC data. While the qualitative behavior of $f_{r}$ vs. H in MgB_2_, mainly the linear decrease with field, is similar to that observed in resonators made of other materials, quantitively, it is very different. The inset in Figure 4 compares $f_{r}\left(H\right)/f_{r}\left(0\right)$ in resonators made of MgB_2_ and YBCO at $T/T_{c}≅1/6$. Evidently, the two materials exhibit drastically different linear slopes, $-9.6\times 10^{-1}$ and $-8\times 10^{-3}$ 1/Tesla for MgB_2_ and YBCO, respectively. In the discussion below, we relate this difference to the different second critical fields in these materials.

**Figure 5:**
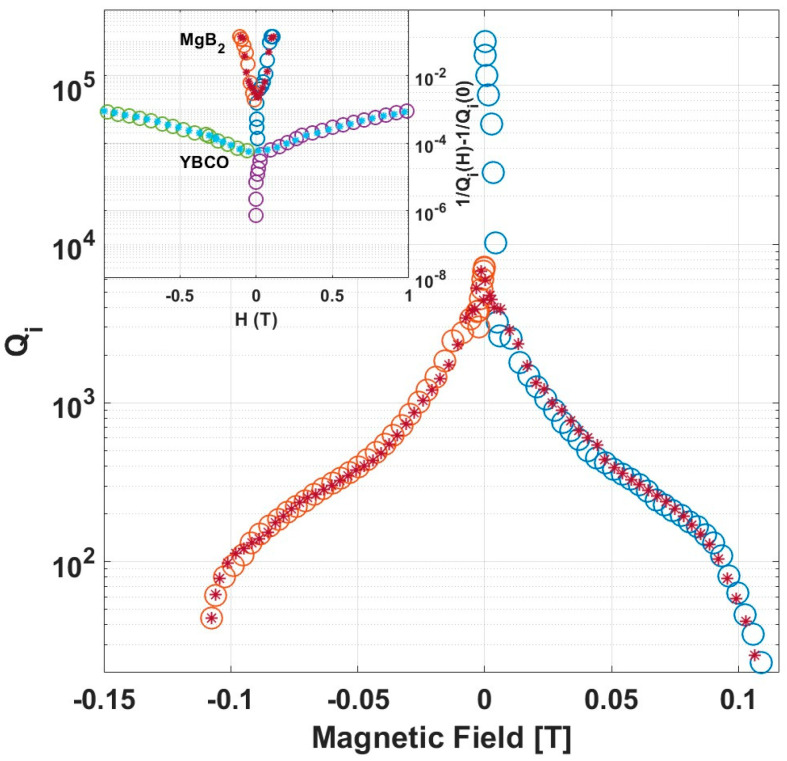
ZFC measurements of the field dependence of Q_i_ at 2.3 K. Color-coding is the same as in Figure 4. Inset: comparison of $\frac{1}{Q_{H}}=\frac{1}{Q_{i}\left(H\right)}-\frac{1}{Q_{i}\left(0\right)}$ vs. field in MgB_2_ and YBCO at $T/T_{c}≅1/6$.

A high sensitivity of $Q_{i}$ to the magnetic field in the MgB_2_ resonator is also exhibited in measurements of $Q_{i}$ vs. H, as shown in the main frame of Figure 5; from a zero-field value of order 10^5^, $Q_{i}$ drops by more than an order of magnitude already at a field of ~0.01 T. As the field increases, it continues to drop moderately in a similar fashion, as observed in the YBCO resonator [8]. The inset in Figure 5 compares the losses due to the magnetic field, $1/Q_{H}=1/Q_{i}\left(H\right)-1/Q_{i}\left(0\right)$, in MgB_2_ and YBCO resonators at $T/T_{c}≅1/6$. Evidently, the losses associated with the field are much more significant in MgB_2_ as compared to YBCO.

In addition to characterizing our MgB_2_ MKID as a resonator, we also characterize it as a photon detector. In measurements of its responsivity and noise equivalent power, we follow the methodology outlined in Refs. [7,24]. The responsivity is calculated as $R_{es}=\Delta {\left|S_{21}\right|}^{2}$/ΔP, where $\Delta {\left|S_{21}\right|}^{2}$ and ΔP denote the change in the power response and the absorbed power of the incident light, respectively. The responsivity was measured for two incident laser powers of 2.66 and 1.88 μW. The power response due to a 1 s pulse was 8 and 6 pW, respectively, yielding responsivities of 3.0 and 3.2 μW/W, respectively. The NEP is calculated as $\frac{P_{n}}{R_{es}\sqrt{\Delta f}}$, where $P_{n}$ is the noise power in a bandwidth of Δf. The noise power at 2 K was about −140 dBm in a bandwidth (BW) of 10 kHz, yielding an NEP of about $3\times 10^{-14}$ and 3.2 × $10^{-14}$ $W/\sqrt{\mathrm{Hz}}$, comparable to the NEP measured in NbN at 4.2 K [25]. To the best of our knowledge, this is the first reported NEP results that have been published on an MgB_2_ MKID. The best NEP reported for an YBCO MKID is 10^−12^ $W/\sqrt{\mathrm{Hz}}$, but this was measured at 10 K. We note that in evaluating the responsivity, we assumed that all the incident laser power is absorbed in the detector, leading to an underestimation of the responsivity; thus, the actual NEP may be even lower than estimated above.

## 4. Discussion

In the following, we show that the origin of the quantitative differences in the temperature and field dependence of the MgB_2_ and YBCO resonators (see insets in Figure 2 and Figure 3) are due to the two-gap nature of MgB_2_ and its lower second critical field, respectively.

The resonance frequency of a half-wavelength superconducting microwave resonator is related to the London penetration depth,$\;\lambda_{L}\left(T\right)$, through the following equation [26]:

(1) $$f_{r}=\frac{1}{2l\sqrt{\left(\frac{\mu_{0}\lambda_{L}^{2}}{A}+L_{m}\right)C}}$$

where A and C are the cross-section area of the resonator and its capacitance per unit length, respectively, $L_{k}=\mu_{0}\lambda_{L}^{2}/A$ and $L_{m}$ are the kinetic and magnetic inductance per unit length, respectively, and l is the resonator length. As shown in Refs. [7,8], the temperature dependence of $f_{r}$ in YBCO can be well described by the temperature dependence of $\lambda_{L}$ as predicted by the two-fluid model:

(2) $$\lambda_{L}=\lambda_{0}{\left(1-{\left(\frac{T}{T_{c}}\right)}^{\gamma }\right)}^{-0.5},$$

with the fitting parameter γ=2.6. At an early stage of MgB_2_ study, it was realized that Equation (2) cannot describe the measured temperature dependence of $\lambda_{L}$ in this material [27,28].

Several authors developed new relations for $\lambda_{L}\left(T\right)$, taking into account the two-gap nature of MgB_2_ [10,29,30]. The best fit to our data was obtained using the expression for $\lambda_{L}\left(T\right)$ given in Ref. [30]:

(3) $$\;\lambda_{L}\left(T\right)=\lambda_{L}\left(0\right){\left(1-2a\sqrt{\frac{2\pi \Delta_{\pi }}{k_{B}T}}e^{-\frac{\Delta_{\pi }}{k_{B}T}}-2\left(1-a\right)\sqrt{\frac{2\pi \Delta_{\sigma }}{k_{B}T}}e^{-\frac{\Delta_{\sigma }}{k_{B}T}}\right)}^{-0.5}$$

where $k_{B}$ is Boltzmann constant, $a=\frac{\lambda_{\sigma }^{2}}{\lambda_{\pi }^{2}+\lambda_{\sigma }^{2}}$, and $\Delta_{\pi }$, $\Delta_{\sigma }$ and $\lambda_{\pi }$, $\lambda_{\sigma }$ are the energy gaps and London penetration depths in the π and σ bands, respectively. Combining Equation (3) with Equation (1), one obtains

(4) $$f_{r}\left(T\right)=f_{r}\left(0\right)\sqrt{1+\chi }{\left({\left(1-2a\sqrt{\frac{2\pi \Delta_{\pi }}{k_{B}T}}e^{-\frac{\Delta_{\pi }}{k_{B}T}}-2\left(1-a\right)\sqrt{\frac{2\pi \Delta_{\sigma }}{k_{B}T}}e^{-\frac{\Delta_{\sigma }}{k_{B}T}}\right)}^{-1}+\chi \right)}^{-0.5}$$

where $\chi =\frac{L_{m}}{L_{k}\left(0\right)},\;L_{m}$ and $L_{k}\left(0\right)$ are the magnetic and kinetic inductance of the resonators, respectively. Using SONNET, following the method described in Ref. [31], we found χ=0.91 for our ${\mathrm{MgB}}_{2}\;$ resonator.

The solid curve in the main frame of Figure 2, calculated using Equation (4) and the parameter values shown in the second column of Table 1, shows a good fit to the data. These parameters are consistent with values reported in the literature for ${\mathrm{MgB}}_{2}$ as listed in the third column of Table 1. The inset in Figure 2, comparing $f_{r}$ vs. $T/T_{c}$ in YBCO and MgB_2_, clearly demonstrates that the two-gap model predicts a faster decrease in $f_{r}$ with temperature as T_c_ is approached.

As mentioned above, the effect of the magnetic field on $f_{r}$, as described in Figure 4, appears qualitatively similar to results previously reported for resonators made of YBCO and other materials. All exhibit an initial sharp decrease in $f_{r}$ in the ZFC data, which has been attributed to the effect of screening currents and a linear behavior thereafter, which has been attributed to the increase in the number of vortices within the resonator [8]. However, the slope $df_{r}/dH$ of this linear decrease differ markedly between resonators made of different materials. This slope can be related to the second critical field, $H_{c2}$, of the material based on the following simplified analysis: The vortex density corresponding to *H_c_*_2_ transforms the entire sample to a normal state. Thus, assuming that the density of vortices is linear with field, the vortex density corresponding to field *H* transforms only a fraction of *H*/*H_c_*_2_ of the sample to a normal state, while the rest is in a superconducting state. Thus, the density of Cooper pairs, $n_{s}$, in field H is given by

(5) $$n_{s}\left(H\right)=n_{s}\left(0\right)\left(1-\frac{H}{Hc_{2}}\right),$$

yielding

(6) $$L_{k}\left(H\right)=\frac{L_{k}\left(0\right)}{1-H/H_{c2}}.$$

For $L_{k}\left(H\right)\gg L_{m}$, one obtains

(7) $$f_{r}\left(H\right)=f_{r}\left(0\right){\left(1-\frac{H}{H_{c2}\;}\right)}^{\frac{1}{2}}≅f_{r}\left(0\right)\left(1-\frac{H}{2H_{c2}}\right)$$

for $H\ll H_{c2}$.

The range of the reported values of $H_{c2}\left(0\right)$ for MgB_2_ and YBCO differ markedly (3–16 T [37,38,39,40] and 50–200 T [41,42,43], respectively). Thus, one would expect also markedly different slopes of $f_{r}$ vs. H for resonators made of these two materials. This is demonstrated in the inset in Figure 4 in which $f_{r}\left(H\right)/f_{r}\left(0\right)$ is plotted versus H. The slopes of these straight lines ($-9.6\times 10^{-1}$ and $-8\times 10^{-3}$ 1/Tesla) yield $H_{c2}$ values at T/T_c_ = 1/6 of 0.52 T and 63 T for MgB_2_ and YBCO, respectively. For YBCO, this value of $H_{c2}$ at T/T_c_ = 1/6 aligns with the values reported in the literature. However, the value of $H_{c2}$ obtained for ${\mathrm{MgB}}_{2}$ is lower than anticipated, a result that needs further investigation.

To explain the behavior of $Q_{i}\left(H\right)$, see Figure 5; we note that according to Equation (5), the lower H_c2_ in MgB_2_ implies a rapid decrease in $n_{s}$ with H. This gives rise to a rapid increase in the density of quasi-particles and thus to a rapid increase in the losses with field. This is demonstrated in the inset in Figure 5 in which the losses due to the field, $1/Q_{H}=1/Q_{i}\left(H\right)-1/Q_{i}\left(0\right)$, are plotted vs. field for MgB_2_ and YBCO. Evidently, the losses in MgB_2_ increase at a much higher rate than in YBCO, reflecting the higher rate of increase in quasi-particles in MgB_2_ due to its smaller $Hc_{2}$.

In summary, exploring the application of MgB_2_ MKIDs was motivated by their relatively high T_c_, which could save the cost and complexity associated with low-temperature cooling systems. Comparing MKIDs made of MgB_2_ and YBCO, we found that MgB_2_ MKIDs are much more sensitive to temperature and external magnetic fields because of their two-gap nature and lower H_c2_. This makes MgB_2_ less attractive in MKID applications as compared to YBCO. However, at low temperatures, below 3 K, MgB_2_ appears to be more advantageous due to its better $Q_{i}$ and NEP. The high $T_{c}$ and low $Hc_{2}$ make the MgB_2_ resonator potentially applicable as a tunable resonator or a magnetic field sensing device that works at relatively high temperature [44,45,46,47].

## Figures and Tables

**Table 1:** Characteristic parameters of ${\mathrm{MgB}}_{2}$ used for calculation of the solid line in Figure 2 compared with a range of reported values [30,32,33,34,35,36,37].

Parameters	$\mathrm{Values}\;\mathrm{Used}\;\mathrm{in}\;\mathrm{Calculation}\;\mathrm{of}\;f_{r}$ (Solid Line in Figure 3)	Range of Values Reported in the Literature
$\Delta_{\pi }$	1.88 meV	1.5–3.5 meV
$\Delta_{\sigma }$	6 meV	6–9 meV
$\lambda_{\pi }$	26 nm	20–40 nm
$\lambda_{\sigma }$	65 nm	40–70 nm

## Data Availability

Data is contained within the article.
